# “The Doctor Needs to Know”: Acceptability of Smartphone Location Tracking for Care Coordination

**DOI:** 10.2196/mhealth.9726

**Published:** 2018-05-04

**Authors:** David T Liss, Eloisa Serrano, Julie Wakeman, Christine Nowicki, David R Buchanan, Ana Cesan, Tiffany Brown

**Affiliations:** ^1^ Division of General Internal Medicine and Geriatrics Northwestern University Feinberg School of Medicine Chicago, IL United States; ^2^ Erie Family Health Center Chicago, IL United States

**Keywords:** delivery of health care, primary care, health information technology, smartphone, mHealth

## Abstract

**Background:**

Care coordination can be highly challenging to carry out. When care is fragmented across health systems and providers, there is an increased likelihood of hospital readmissions and wasteful health care spending. During and after care transitions, smartphones have the potential to bolster information transfer and care coordination. However, little research has examined patients’ perceptions of using smartphones to coordinate care.

**Objective:**

This study’s primary objective was to explore patient acceptability of a smartphone app that could facilitate care coordination in a safety net setting. Our secondary objective was to identify how clinicians and other members of primary care teams could use this app to coordinate care.

**Methods:**

This qualitative study was conducted at a federally qualified health center in metropolitan Chicago, IL. We conducted four focus groups (two in English, two in Spanish) with high-risk adults who owned a smartphone and received services from an organizational care management program. We also conducted structured interviews with clinicians and a group interview with care managers. Focus groups elicited patients’ perceptions of a smartphone app designed to: (1) identify emergency department (ED) visits and inpatient stays using real-time location data; (2) send automated notifications (ie, alerts) to users’ phones, asking whether they were a patient in the hospital; and (3) send automated messages to primary care teams to notify them about patients’ confirmed ED visits and inpatient stays. Focus group transcripts were coded based on emergent themes. Clinicians and care managers were asked about messages they would like to receive from the app.

**Results:**

Five main themes emerged in patient focus group discussions. First, participants expressed a high degree of willingness to use the proposed app during inpatient stays. Second, participants expressed varying degrees of willingness to use the app during ED visits, particularly for low acuity ED visits. Third, participants stated their willingness to have their location tracked by the proposed app due to its perceived benefits. Fourth, the most frequently mentioned barriers to acceptability were inconveniences such as “false alarm” notifications and smartphone battery drainage. Finally, there was some tension between how to maximize usability without unnecessarily increasing user burden. Both clinicians and care managers expressed interest in receiving messages from the app at the time of hospital arrival and at discharge. Clinicians were particularly interested in conducting outreach during ED visits and inpatient stays, while care managers expressed more interest in coordinating postdischarge care.

**Conclusions:**

High-risk primary care patients in a safety net setting reported a willingness to utilize smartphone location tracking technology to facilitate care coordination. Further research is needed on the development and implementation of new smartphone-based approaches to care coordination.

## Introduction

Care coordination—the deliberate organization of information transfer and care processes to facilitate the appropriate delivery of health services—is a pillar of high-functioning primary care practices [1,2], but can be highly challenging to carry out. In many communities, such as large urban areas, care is fragmented across health systems and providers [3,4]. Resulting failures in care coordination can lead to adverse outcomes such as hospital readmissions [5] and wasteful health spending [5,6].

Health information technology (IT) and targeted chronic care interventions can support care coordination, but the reach and effectiveness of these approaches is limited. Electronic health records (EHRs) are often unable to facilitate care coordination between organizations [4,7], and less than half of US hospitals [8] and providers [9] participate in an operational health information exchange (HIE). While interventions that prioritize care coordination, such as disease management and transitional care programs for chronically ill patients, have reduced hospital admissions [10-12] or improved patient-reported outcomes [11,12], their replicability and scalability are limited by a lack of global cost-savings [10,13]. New, high-value approaches to care coordination are urgently needed.

Smartphones are increasingly ubiquitous among adults [14,15] and have the potential to bolster information transfer and care coordination in health care. For example, if a patient is discharged from an inpatient stay, smartphone location tracking technologies might be able to identify the discharge in real time, and then send a message to the patient’s primary care provider (PCP) to initiate postdischarge care coordination. Although one prior study evaluated the use of smartphone-based geofencing (ie, the creation of virtual geographic boundaries to define a particular location) to ascertain hospitalizations [16], to our knowledge no prior research has examined patients’ perceptions of using smartphones to coordinate care.

The primary aim of this study was to explore patient acceptability of a smartphone app that could facilitate care coordination in a safety net setting. Our secondary aim was to identify how clinicians and other members of primary care teams could use this app to coordinate care. We hypothesized that the proposed app would be acceptable to patients if it was minimally disruptive and easy to use.

## Methods

### Study Design and Setting

This qualitative study was conducted at Erie Family Health Center (described henceforth as “Erie”), a federally qualified health center (FQHC) in metropolitan Chicago, IL that serves a largely Hispanic patient population; 73% of Erie patients are Hispanic, and 47% are best served in Spanish [17]. Between November 2015 and January 2016, we conducted four patient focus groups (two in English, two in Spanish), structured interviews with clinicians, and a group interview with care managers. All study protocols were approved by Northwestern University’s Institutional Review Board.

Erie owns and operates seven adult primary care clinics, all of which have achieved Joint Commission Primary Care Medical Home certification. The majority of Erie’s patient population is low income, over 80% are racial/ethnic minorities, and approximately 60% have Medicaid coverage [17]. Since 2014, Erie has had a care management program for high-risk patients; this program is funded through an Accountable Care Organization and a Medicaid Managed Care Organization that was established during Illinois’ Medicaid expansion under the Affordable Care Act [18]. Care managers are colocated with clinicians at each clinic and work with patients on tasks such as chronic care planning and coordination of care following emergency department (ED) visits and inpatient stays. Patients screen into the care management program through criteria such as repeated ED or inpatient use, inpatient stays for chronic illnesses, or the presence of numerous social and clinical risk factors.

Erie also partners with several local organizations to support coordination of care. Erie clinicians have read-only access of EHRs at some local hospitals and specialty care providers. Erie providers can also access real-time data on the use of several local EDs for patients in one local Medicaid plan. However, Chicago does not have an operational citywide HIE [19,20], and Erie providers cannot comprehensively detect hospital use across the Chicagoland metro area.

### Participants

Patients were eligible for focus group participation if they were: an adult (age 18-89) whose preferred language was English or Spanish; receiving services from an Erie care manager; and self-reported ownership of a smartphone. Patients were excluded if they were pregnant, or they had dementia or another behavioral health condition where Erie staff felt it would be inappropriate to contact them. We recruited patient participants by mailing them a lead letter about the study and a number to call to opt out of recruitment. Approximately one week later, a bilingual Erie research assistant called patients to tell them about the study and screen for study eligibility. Eligible, interested patients who elected to participate in the study provided written informed consent at the beginning of each in-person focus group; participants received a US $30 gift card at the end of each focus group, which averaged approximately 90 minutes.

Clinicians were recruited for individual phone interviews via convenience sampling. The group interview included all attendees at a monthly meeting for Erie’s care management program. Clinicians and care managers voluntarily participated in interviews and provided informed consent at the beginning of each interview.

**Figure 1:**
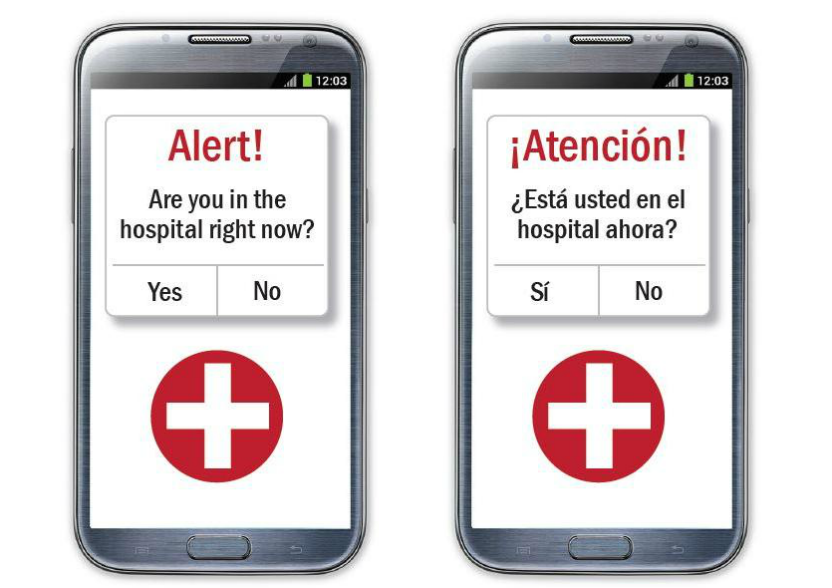
Proposed push notification sent to patient’s smartphone after app detects a potential hospital visit (left: English; right: Spanish).

### Interview Guide and Data Collection

Prior to each focus group, patient participants completed a brief questionnaire about their demographic characteristics, comorbidities, and smartphone usage. The moderator then used a semistructured guide to elicit participants’ perceptions of a smartphone app designed to: (1) use real-time smartphone location data to identify potential ED visits and inpatient stays at Chicago-area hospitals; (2) send automated push notification s (ie, alerts) to users’ phones, asking them whether they were a patient in the hospital, such as the messages presented in Figure 1 (image on the left presented at English focus group; image on the right presented at Spanish focus group); and (3) send automated messages to Erie primary care teams to notify them about patients’ confirmed hospital visits.

The moderator asked participants about their willingness to respond to push notifications from the app. Participants were also asked about their preferred wording and frequency of alerts, their perceptions of “false alarms” (ie, push notifications sent at times when they were not receiving emergency or inpatient care), as well as potential privacy concerns and desirable/undesirable app features.

Clinicians and care managers were asked about the timing and modality (eg, secure message, telephone call) of automated messages they would like to receive from the app, data points to include in messages, and how to integrate messages into existing clinical workflows. Patient-facing study documents were drafted in English and translated into Spanish by a certified translation service. Clinician and care manager interviews were conducted in English. All focus groups and interviews were audio recorded and transcribed.

### Analysis

Patient focus group transcripts were analyzed by two members of the research team (DTL and JW analyzed English transcripts; ES and KN analyzed Spanish transcripts) in multiple rounds to organize the content into emergent themes [21,22], where we derived basic concepts (themes) from the data and compared them with other data to facilitate meaningful categorizations [23,24]. After an initial round of analysis by each coder, coders met to generate a list of common thematic categories across focus groups. In the second round of analyses, each coder assigned theme-based codes to the qualitative results, and discrepancies between coders were addressed based on further discussion and consensus within each coding dyad. See Multimedia Appendix 1 for a complete list of themes and codes. Coders then reviewed the transcripts together and selected quotes that exemplified major themes. Clinician and care manager responses to interview questions were reviewed and summarized by two members of the research team (DTL and JW).

## Results

### Patient Focus Groups

The four patient focus groups took place in November and December 2015, with 16 patients participating. Baseline characteristics are shown in Table 1. Most participants were aged either 30-49 or 50-64 years, the majority were female, and half were Latino/Hispanic. As might be expected of patients under active care management, there were high rates of self-reported chronic illness, with seven (7/16, 44%) reporting diabetes, four (4/16, 25%) reporting asthma, and eight (8/16, 50%) reporting hypertension. Nine of 16 participants (56%) reported owning smartphones with the Android operating system, and only one reported owning a smartphone with the iOS operating system. One participant had a Windows phone, and five (5/16, 31%) reported other/missing operating system.

Five main themes emerged in patient focus group transcripts (Table 2). First, participants expressed a high degree of willingness to use the app and respond to push notifications during inpatient stays. Participants felt that the app would keep their PCP informed about important developments in their care, which would in turn promote communication with the PCP during the inpatient stay or soon after discharge. When asked about their willingness to respond to the proposed push notifications after being admitted to the hospital, one participant stated, “Well, yeah, the doctor needs to know.”

Second, patient participants expressed varying degrees of willingness to use the app during ED visits, particularly for low acuity events. Some participants described using the ED as an alternative source of primary care or a source of after-hours primary care. Selected participants, some of whom were relatively new patients at Erie and may have been uninsured prior to the Affordable Care Act Medicaid expansion, stated that they might respond “No” to a push notification if they were in the ED for a nonsevere condition.

Third, participants stated their willingness to have their location tracked by the proposed app, due to its perceived benefits; they had prior experience with location tracking and seemed to accept it as a part of smartphone ownership and modern society. As one participant stated, “We’re being followed and watched every day, all day long so what’s the problem with a[n] app locating where you at?” Perhaps most importantly, participants implied or explicitly stated that the app was serving a desirable function and they understood how location tracking was being used to achieve this goal.

**Table 1:** Participant characteristics for patient focus groups (N=16).

Characteristic		n (%)
**Age (years)**		
	18-29	3 (18.8)
	30-49	6 (37.5)
	50-64	6 (37.5)
	+65	1 (6.2)
**Sex**		
	Female	9 (56.2)
	Male	7 (43.8)
**Race/ethnicity**		
	Non-Hispanic white	2 (12.5)
	Non-Hispanic black/African American	6 (37.5)
	Hispanic/Latino	8 (50.0)
**Chronic illnesses**		
	Diabetes	7 (43.8)
	COPD^a^, chronic bronchitis or emphysema	1 (6.2)
	Asthma	4 (25.0)
	Hypertension	8 (50.0)
	Coronary artery disease	0 (0)
	Heart failure	1 (6.2)
**Smartphone operating system**		
	Android	9 (56.3)
	iOS	1 (6.2)
	Windows Phone	1 (6.2)
	Other/missing	5 (31.3)

^a^COPD: chronic obstructive pulmonary disease.

**Table 2:** Emergent themes from qualitative analysis of patient focus groups.

Theme	Related quotes
Acceptable overall, willing to use app during inpatient stays	I think what’s good about it is to let him know that I’m in the hospital now and come see you soon, because evidently something really seriously happened to me to be in the hospital and to be, like you said, to be sitting in there in a hospital bed. So of course I would want him to know. I think that would be a good app, to let him know. It’s easy, it’s one button.^a^
Limited willingness to use app during low-acuity emergency department visits	I don’t want to bother my doctor with the fact that nobody could get me in, but my fever’s 101, I think I need an antibiotic but it’s not an emergency but my doctor couldn’t see me until next Thursday. So I say, “No,” I’m not actually here, even though I’m here. If I am in the emergency room, I think it would be [good to respond] after you are admitted and they tell you what you have.^a^
Willingness to have location tracked to share important information	I think that it’s really good that it does know where you’re at. Facebook has [location tracking], Google has it, Twitter has it, Instagram has it. Everything has your information. So, to have something that is necessary, something important, it won’t bother me. If all apps have my information, it won’t bother me to have one more.^a^
Barriers to acceptability	The location - the number one thing for me is I am not going to download the app if it is completely going to drain my battery. If it’s just going to use up my whole entire battery from running in the background, I’m not going to want it on my phone and I think a lot of people would agree. If like you said, every time I pass the front of the hospital, I receive an alarm, I’d rather delete it.^a^
Usability: tension between adding features and increasing user burden	If it’s your app, okay, you [have] got to make it personal because if it’s your app, you know, you see apps sometimes, they say hello [name], or hello so and so, it should know that it’s you. See what I’m saying? So maybe when you first get it you would put your name on there and everything. When that alert comes on it’s going to say [name], are you in the hospital? Are you in the emergency room? Well I think the correct thing would be for [my doctor] to know exactly why they are seeing you.^a^ The only thing [my doctor] needs to know is that you’re in the hospital and after you’re out you can go to her and take all the paperwork or she asks you why you were in.^a^

^a^Quote from Spanish-speaking participant translated into English.

Fourth, inconveniences to app users were the most frequently mentioned barriers to acceptability. Participants did not want to receive “false alarm” notifications if they were not in the hospital, and they thought that push notifications should not fire too soon after they entered a hospital building. If they initially did not respond to push notifications, they did not want these notifications to repeat too frequently, since their initial failure to respond could signify that they were unavailable due to factors such as being unconscious or asleep. One participant stated that other apps with location tracking functions had drained his phone’s battery, and that he was unwilling to use any apps with this flaw.

Finally, there was some tension between how to maximize usability without unnecessarily increasing user burden. On one hand, a limited number of participants said they would like to use the app to send clinical information about their hospital visit (ie, their admitting diagnosis or which inpatient unit they were in) to their primary care team. Some participants also expressed a desire to personalize the app through features such as customizable app settings (eg, tailored push notification sounds) or including their name in push notifications. However, there was not a high degree of agreement between participants about which clinical data points to share with primary care teams, or about the most desirable features to include in the app.

One area of participant agreement was the confusing wording of the question in proposed push notifications (Figure 1), which asked app users whether they were in the hospital, without distinguishing between patients receiving hospital-based medical care and other hospital visitors, such as those visiting a hospitalized friend or family member. In one focus group, several participants agreed that this question should be changed to, “Are you a patient in the hospital right now?”

### Clinician/Care Manager Interviews

Three clinicians (two physicians, one nurse practitioner) participated in phone interviews. In the care management group interview, nine respondents (seven care managers, the care management program coordinator, and the program manager) participated. Clinicians and care managers expressed interest in receiving messages from the app at both the patients’ time of hospital arrival and at discharge. Clinicians were particularly interested in acting on messages received at arrival in order to conduct outreach to the emergency/inpatient care team, while care managers expressed more interest in acting on alerts received at discharge in order to coordinate postdischarge care. Respondents were interested in obtaining automated data from the app, such as hospital name, phone number, and the patient’s approximate time of arrival and discharge. Clinicians expressed interest in receiving EHR-based alerts (ie, flags) about patients’ hospital arrival during times when they were seeing patients in clinic; they were interested in receiving text messages at other times. Care managers were most interested in receiving EHR-based alerts about hospital arrival and discharge.

Some clinicians and care managers expressed interest in obtaining clinical information that would require manual data entry by app users, such as admitting diagnosis, discharge diagnosis, or changes in medication regimens. However, these sentiments were counterbalanced by a desire to limit both the number of messages sent to care teams and the extent to which patients would expect real-time responses from their care teams.

## Discussion

### Principal Results

In these focus groups, high-risk primary care patients reported a willingness to utilize smartphone location tracking technology to facilitate care coordination. Notably, study participants already received care coordination services through their FQHC’s care management program, yet they saw value—and opportunities for potential improvement—in the proposed smartphone-based approach to facilitating information transfer and care coordination. Participants were particularly willing to respond to push notifications about inpatient stays, although some expressed limited willingness to use the app to notify care teams about low-acuity ED visits. Participants expressed concern about barriers to acceptability, such as “false alarm” notifications and draining their phone’s battery. While usability might be increased by allowing users to tailor the look and feel of the app, there was a lack of agreement—and the potential for decreased usability—about data that should be manually entered into the app.

### Comparison With Current Evidence

These results are informative in the context of the current health IT landscape and may represent an opportunity for developing new approaches to care coordination. The inability to transfer clinical information between organizations remains a persistent barrier to care coordination, as evidenced by a recent decrease in the number of operational HIEs [9] and the challenges primary care practices face in using health IT to coordinate emergency and inpatient care [4]. The potential benefits of the proposed app may be especially high in regions with no HIEs, particularly in population centers with multiple hospitals. The proposed app may also have high utility in settings where information transfer is fragmented during care transitions, such as small outpatient practices that are not affiliated, or lack formal information sharing protocols, with local hospitals.

Our findings seem to align with those of a prior qualitative study, in which consumers expressed willingness to make tradeoffs between privacy and security if a mobile health (mHealth) intervention offered increased convenience or benefits [25]. Additionally, given that over three-fourths of American adults own smartphones [15], the potential scalability of the app proposed in these focus groups is extremely high.

These findings are also noteworthy in the context of health IT use in safety net settings and among racial/ethnic minorities. Although minorities are disproportionately likely to own a smartphone [14] and to have chronic conditions requiring care coordination [26,27], past research has found that racial/ethnic minorities had lower uptake of technologies such as patient portals [28,29] or a longitudinal mHealth intervention with daily text messaging [30]. In contrast, high-risk FQHC patients in this study expressed a willingness to use the proposed app, which leverages automated location tracking technologies in a way that could enhance information transfer to PCPs, while requiring minimal human effort from app users.

### Limitations

This study has several limitations of note. The smartphone app proposed in these focus groups may be unnecessary in settings such as communities with operational HIEs, or in integrated delivery systems with well-organized care coordination workflows between the primary care, emergency, and inpatient settings. This was an exploratory study that was conducted in a single urban FQHC, and our findings may therefore not be generalizable to other organizations or populations. Further research on the proposed app is needed in other populations and settings (eg, privately insured patients, non-FQHC sites) to examine the external validity of our findings. Additionally, the study sample consisted of high-risk patients in a care management program who owned smartphones; these participants may have been more knowledgeable about care coordination, and more motivated to address current gaps in information transfer, compared to lower-risk groups or patients who lack a source of comprehensive, team-based primary care. Individuals who voluntarily participate in focus groups about a smartphone app may also be more comfortable with health IT than the general population. By design, we only held a small number of focus groups and interviews at this early stage of the app development process. This small sample size was sufficient for addressing our study aims but may further limit the generalizability of findings. Unfortunately, nearly one third of focus group participants did not provide data on their smartphone’s operating system. However, most of those who provided data for this questionnaire item had an Android phone; this finding is consistent with published national data showing much higher rates of Android ownership (compared to iOS) among low income groups [31]. Early app development efforts in the patient population under study here should therefore prioritize an Android-based app, followed by an iOS app.

### Conclusions

A proposed smartphone-based approach to facilitating care coordination was acceptable to high-risk adults in an urban FQHC. Further research is now needed on the feasibility of developing and implementing this type of smartphone app within an organizational care coordination initiative, and its potential effects on information transfer and care coordination.

## Appendix

Multimedia Appendix 1 List of themes and codes from analysis of patient focus group transcripts.

## Abbreviations

ED: emergency department
EHR: electronic health record
FQHC: federally qualified health center
HIE: health information exchange
IT: information technology
mHealth: mobile health
PCP: primary care provider
