# Supplementary material for: “The Doctor Needs to Know”: Acceptability of Smartphone Location Tracking for Care Coordination
Source: JMIR Mhealth Uhealth. 2018 May 4;6(5):e112. doi: 10.2196/mhealth.9726 (PMC5960037; doi:10.2196/mhealth.9726)
Supplement: Multimedia Appendix 1 [file mhealth_v6i5e112_app1.pdf]

Multimedia Appendix 1: List of Themes and Codes from Analysis of Patient Focus Group Transcripts

**Theme**	**Codes**
[A] Overall acceptability of app	[A1] Yes/willing to use
	[A2] No/unwilling to use
[B] Advantages of prompts	[B9] General comments about advantages
[C] Disadvantages of prompts	[C1] False alarms
	[C2] Draining battery
	[C3] Timing/number of prompts
	[C4] Cell coverage in hospital
	[C5] Unable to respond, incapacitated in hospital
[D] Prompt design	[D1] Visual look
	[D2] Wording of prompts
	[D3] Personalization of prompts (e.g. why at hospital)
	[D4] Allow delayed response (e.g. snooze button)
	[D5] Prompt sounds/vibrations
	[D6] Timing of prompts
[E] Prompts for emergency department (ED) visits: willingness to respond	[E1] Yes/would respond to prompt
	[E2] Depending on situation (e.g. how sick you are)
	[E3] No/undesirable
[F] Optional features	[F1] Alerts to family/friends
	[F2] Alerts to non-Erie providers
	[F3] Medication reminders and/or other app functions
[G] Privacy/location tracking concerns	[G1] Yes, privacy concerns
	[G2] No privacy concerns
	[G3] Yes, concerns with data breach/hacking
	[G4] No concerns with data breach/hacking
[X] Other	[X9] Other
